# Biomechanical effects of supplemental posterior cervical stabilization in one-to three-level anterior cervical discectomy and fusion: a finite element analysis of construct length–dependent stress shielding and adjacent segment mechanics

**DOI:** 10.3389/fbioe.2026.1898142

**Published:** 2026-07-29

**Authors:** Junwei Zhang, Tairui Zhang, Maji Sun, Yang Sun, Shuo Feng, Jun Sun, Feng Yuan

**Affiliations:** 1 Department of Orthopedics, The Second Affiliated Hospital of Xuzhou Medical University, Xuzhou, China; 2 Department of Orthopedics, Affiliated Hospital of Xuzhou Medical University, Xuzhou, China

**Keywords:** adjacent segment degeneration, anterior cervical discectomy and fusion, biomechanics, cervical spine, finite element analysis, posterior cervical fixation

## Abstract

**Objective:**

This finite element analysis study aimed to compare the biomechanical behavior of anterior cervical discectomy and fusion (ACDF) with circumferential cervical fusion (CCF) across one-, two-, and three-level constructs, to elucidate the length-dependent contribution of supplemental posterior fixation.

**Methods:**

A C3-C7 finite element model was developed from CT scans. Seven scenarios were simulated: intact spine; 1-, 2-, and 3-level ACDF; and corresponding CCF constructs (ACDF plus bilateral posterior facet cages). A 73.6 N axial load with ±1.0 Nm moments simulated flexion, extension, lateral bending, and axial rotation. Stress on vertebrae, instrumentation, and adjacent segments (C3/4, C5/6, C6/7 facet capsules and discs) was analyzed.

**Results:**

The biomechanical benefit of CCF was length-dependent. It reduced anterior plate stress relative to ACDF, with increasing reduction for longer constructs (8%–12% for 1-level, 10%–18% for 2-level, 15%–25% for 3-level). CCF provided greater vertebral stress shielding. All fusions increased adjacent facet capsule stress, slightly more with CCF. However, CCF resulted in lower increases in adjacent disc stress than ACDF (e.g., at C6/7 disc: +1.6% for 3-level CCF vs. +5.8% for ACDF).

**Conclusions:**

One-level ACDF provides sufficient stability. For two-level constructs, posterior fixation offers measurable protection, beneficial for high-risk patients. For three-level constructs, CCF effectively mitigates the significant anterior stress concentration and elevated adjacent disc stress associated with standalone ACDF, despite a minor increase in facet capsule stress. Posterior augmentation should be considered based on construct length.

## 1 Introduction

ACDF remains the leading surgical treatment for cervical radiculopathy and myelopathy, the NSQIP database from 2012 to 2022 shows that ACDF accounted for 90.2% of cervical spine surgeries during the same period (Robertson and Ashley, 2023; Tse et al., 2025). For one- and two-level degenerative disc disease, ACDF consistently gives high fusion rates (typically >90%) and good clinical results (Gökoğlu et al., 2025; Mun et al., 2025). But when three or more contiguous levels are affected, the biomechanical demands on the anterior construct rise sharply (Liu et al., 2025; Mumtaz et al., 2022). Longer ACDF constructs have significantly higher rates of pseudarthrosis, adverse events, and revision surgery compared to shorter ones (Choi et al., 2017; Steinhaus et al., 2020; Wewel et al., 2019).

Multilevel anterior cervical discectomy and fusion (ACDF) presents biomechanical challenges due to the extended lever arm and increased cumulative stress at bone-implant interfaces, particularly at the lower end of the construct (Huang et al., 2022; Razzouk et al., 2023). To mitigate these challenges, some surgeons have adopted circumferential cervical fusion (CCF), a technique that combines ACDF with additional posterior cervical fusion (PCF) (Gallagher et al., 2024; Kramer et al., 2020). By introducing more fixation points and limiting motion at the treated levels, CCF aims to enhance construct stability and facilitate fusion (Bakare et al., 2023). The additional surgical challenges, including prolonged operating time, increased blood loss, and potential morbidity associated with the posterior approach, must be weighed against the anticipated biomechanical improvements (Koller et al., 2009).

A recent multicenter randomized trial by Strenge et al. (2025) compared three-level ACDF alone with CCF using an investigational posterior stabilization system (PCSS). CCF significantly improved 12-month fusion success (61% vs. 17%, P < 0.001) and lowered 24-month revision rates (2% vs. 23%, P < 0.001) without raising adverse events. Those clinical findings strongly support adding posterior fixation for three-level disease, but the underlying biomechanical reasons are not fully understood. Specifically, it is not known whether the biomechanical advantages of CCF apply to all construct lengths and become more significant as more segments are fused.

Finite element analysis (FEA) is highly effective for investigating these issues, as it enables systematic evaluation of stress distribution, structural stability, and the mechanics of adjacent segments under controlled experimental settings. Unlike clinical studies, FEA cannot isolate surgical technique effects from patient-specific factors like bone quality and healing capacity. Parametric analyses of varying construct lengths can pinpoint the thresholds at which ACDF alone fails to meet biomechanical requirements.

Therefore, we did a whole FEA to compare ACDF and CCF for one, two, and three-level constructs. Our objectives included: (1) detailing the length-dependent effects of ACDF and CCF on vertebral stress, implant stress, and adjacent segment mechanics; (2) exploring the protective mechanisms associated with posterior fixation; and (3) determining the specific construct length at which CCF offers a significant biomechanical advantage. Our findings seek to provide a mechanism that supports clinical evidence and could guide surgical decisions for patients with multilevel cervical degenerative disease.

## 2 Materials and methods

### 2.1 Model construction

A three-dimensional finite element model of the cervical spine, spanning from C3 to C7, was constructed utilizing CT scans obtained from a healthy adult male with no prior cervical spine pathology (Wheeldon et al., 2008). The CT images had a slice thickness of 0.625 mm, and were imported into Mimics Medical 21.0 (Materialise, Belgium) to segment cortical bone, cancellous bone, and endplates. The segmented geometry was subsequently exported to Geomagic Studio 2014 (64-bit version; 3D Systems, United States) for further processing, including surface refinement, smoothing, and NURBS surface generation, resulting in a solid model with precise anatomical accuracy. This solid model was imported into SOLIDWORKS 2024 (Dassault Systèmes, United States) for assembly and surgical simulation. Intervertebral discs are composed of the nucleus pulposus and annulus fibrosus. The study incorporated five primary ligaments—anterior longitudinal, posterior longitudinal, ligamentum flavum, interspinous, and capsular ligaments—modeled as nonlinear spring elements, with stiffness coefficients sourced from existing literature (Panjabi et al., 2001; Zhang et al., 2006).

### 2.2 Surgical simulation

We created simulations of seven surgical scenarios to facilitate systematic comparisons of different construct lengths and techniques: (1)Intact model: C3–C7 is the baseline; (2) One level ACDF: discectomy and fusion at C4/5 with anterior plate screw construct and interbody cage; (3) One level CCF: one level ACDF at C4/5, and bilateral posterior facet cages at the same level; (4) Two-level ACDF: discectomy and fusion from C4 to C6 with anterior hardware; (5) Two level CCF: two level ACDF plus posterior facet cages at C4/5 and C5/6; (6) Three-level ACDF: discectomy and fusion from C3 to C6 with anterior hardware; (7) Three level CCF: three level ACDF plus posterior facet cages at C3/4, C4/5, and C5/6.

**FIGURE 1:**
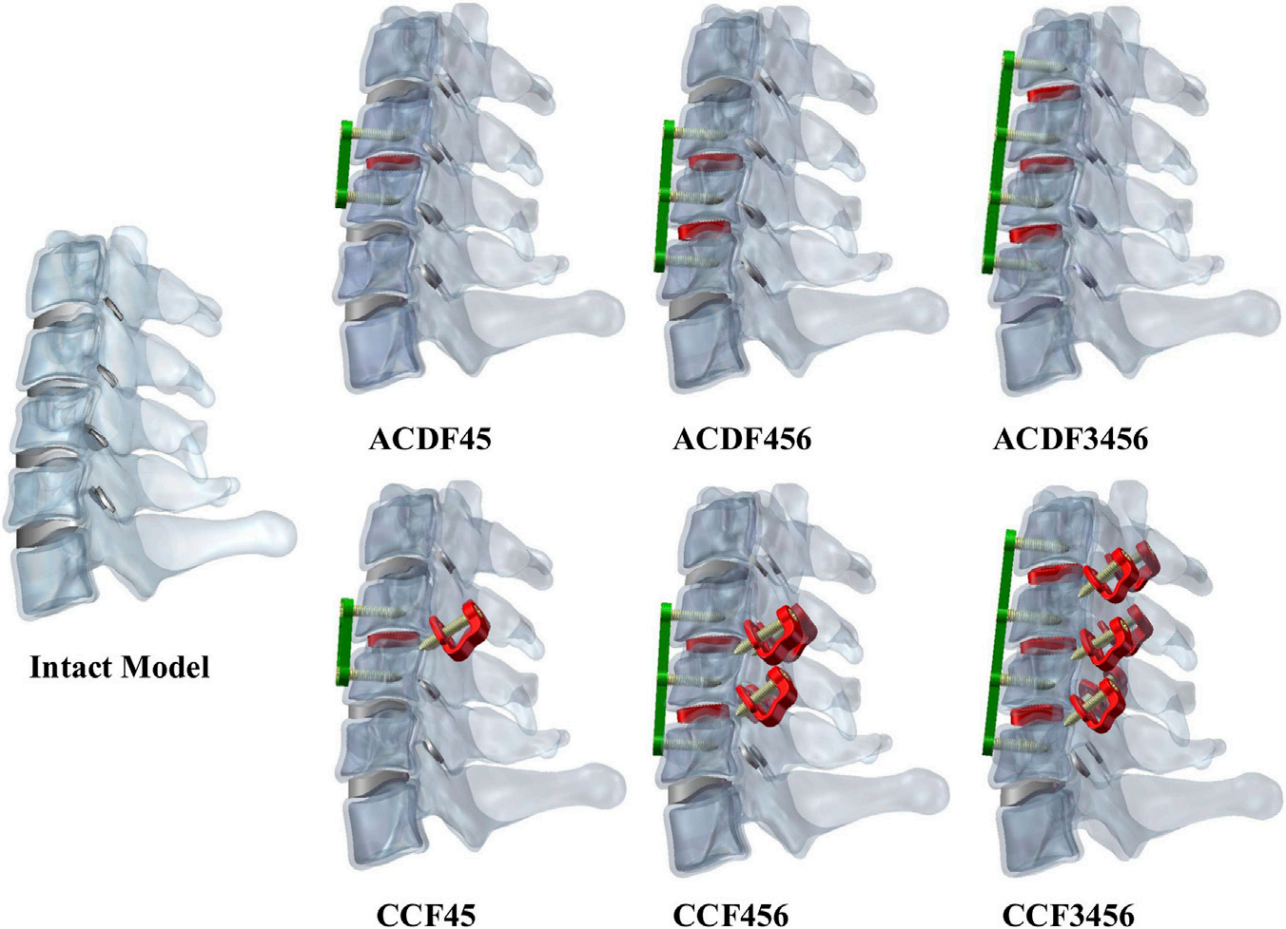
The model diagram presents a C3–C7 finite element mesh and surgical constructs.

All the anterior constructs employed a semiconstrained titanium plate with bicortical screws (4.0 mm diameter, 14 mm length) and PEEK interbody cages (Ahn et al., 2023). Posterior facet cages were modeled as titanium implants with integrated fixation screws, and they were placed bilaterally into the facet joints according to the manufacturer’s specifications (Pereira et al., 2021). All the implants were provided with accurate geometry and material properties (see Figure 1) (Tan et al., 2022).

### 2.3 Material properties and mesh generation

We assigned linear elastic, isotropic material properties to all structural components, based on the values from earlier validated cervical spine FEA studies7 (Table 1). Ligaments were modeled as a tension-only spring element with nonlinear stiffness.

**TABLE 1:** Characteristics of spinal component materials and associated instrumentation.

Component	Elastic modulus (MPa)	Poisson’s ratio	Spring stiffness (N/mm)
Cortical bone	12,000	0.29	–
Cancellous bone	450	0.29	–
Endplate	500	0.40	–
Nucleus pulposus	1	0.49	–
Annulus fibrosus	3.4	0.40	–
Facet cartilage	10.4	0.49	–
Titanium Ti-6AI-4V	110,000	0.30	–
Interbody cage (PEEK)	3,600	0.30	–
Capsular ligament	–	–	0.52
Anterior longitudinal ligament	–	–	4.0
Posterior longitudinal ligament	–	–	2.5
Ligamentum flavum	–	–	0.86
Interspinous ligament	–	–	0.71

HyperMesh 2021 (Altair Engineering, United States) was used for mesh generation. All osseous structures, interbody cages, and plate-screw constructs were meshed using 10-node tetrahedral elements (SOLID187). The annulus fibrosus and nucleus pulposus were meshed using 8-node hexahedral elements (SOLID185) with reduced integration to avoid shear locking. Ligaments were modeled as tension-only 2-node spring elements (COMBIN39) with nonlinear stiffness properties. A convergence test was employed with mesh sizes of 2.5, 2.0, 1.5, and 1.0 mm. Since the von Mises stress variation between successive refinements remained below 5% at a size of 1.5 mm, this dimension was selected as the optimal balance between precision and computational efficiency (Lin et al., 2023). Table 2 provides the final tally of elements for each model.

**TABLE 2:** Mesh information for each surgical model.

Model	Nodes	Elements
Intact model	473,698	287,709
ACDF 45	629,228	373,260
CCF 45	721,686	424,176
ACDF 456	721,631	425,500
CCF 456	912,932	531,330
ACDF 3456	1,021,201	623,413
CCF 3456	1,242,762	742,379

### 2.4 Boundary conditions and loading protocol

**FIGURE 2:**
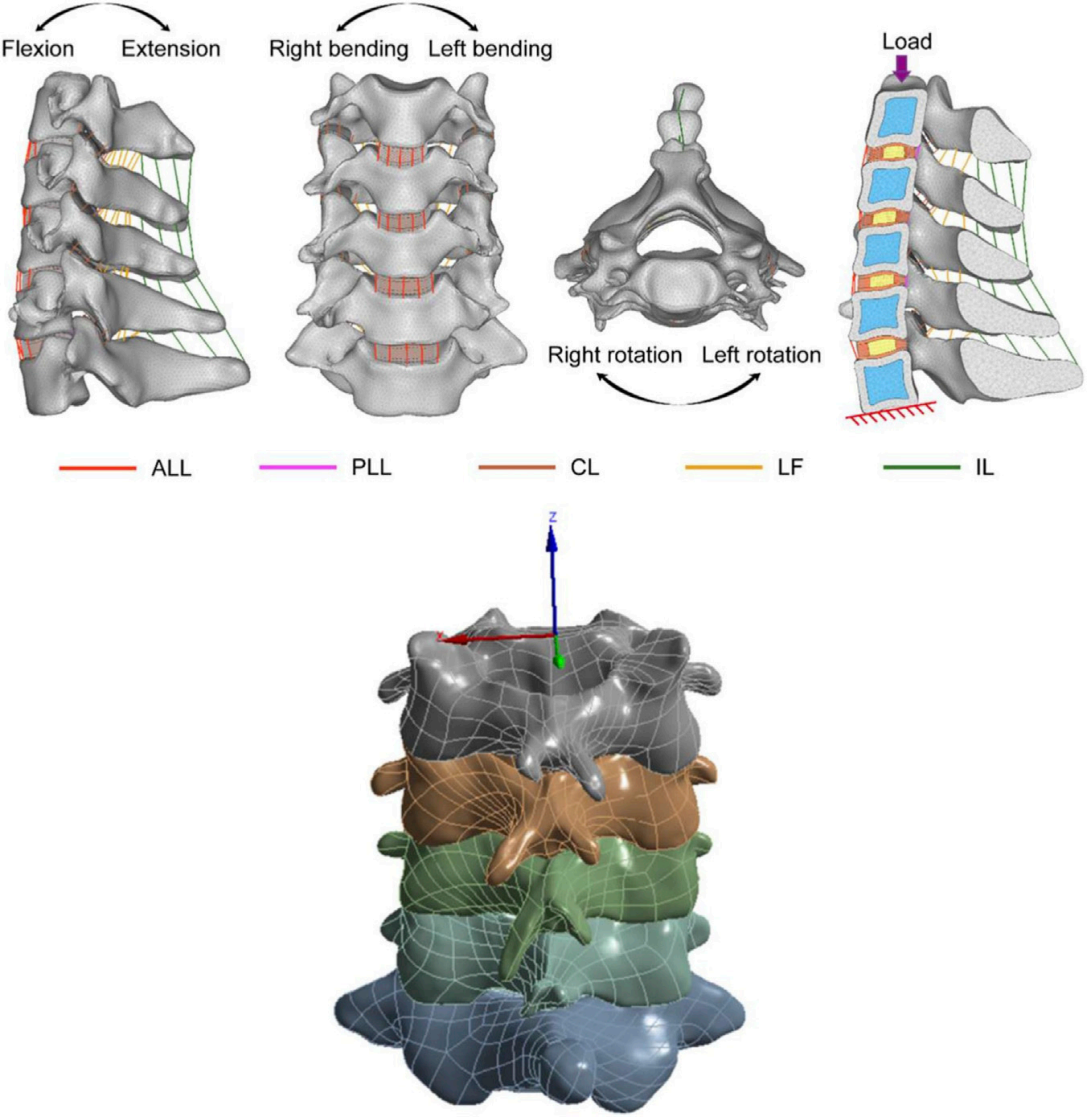
Load the coordinate system settings. Fx = 0, Fy = 0, Fz = 73.6 N (simulating head weight); Mx = ±1 Nm, My = ±1 Nm, Mz = ±1 Nm (simulating flexion/extension, lateral bending, and axial rotation, respectively).

Contact interactions between components were defined as follows: bone-implant interfaces (including screw-bone, cage-endplate, and facet cage-bone interfaces) were modeled as bonded contacts (no relative motion) to simulate immediate postoperative rigid fixation (Lee et al., 2023). The facet cartilage surfaces were modeled as frictionless contacts to allow physiologic sliding motion. The interface between the nucleus pulposus and annulus fibrosus was defined as bonded to ensure continuous load transmission. All spring elements representing ligaments were attached to bone via shared nodes at their insertion sites. We adjusted the surface of C7 in all degrees of freedom. A 73.6 N axial compressive load was applied to the top of C3 to simulate head weight. In the three anatomical planes, pure moments of ±1.0 Nm were applied to produce flexion, extension, lateral bending, and axial rotation (Ke et al., 2021; Liang et al., 2022) (Figure 2). All loads were applied quasi-statically, and we calculated the kinematics and stress distributions with ANSYS Workbench 2021 R^2^ (ANSYS, Inc., United States).

### 2.5 Outcome measures

For each model and loading condition, the following outcomes were extracted and analyzed: vertebral body stress, assessed via von Mises contour maps and peak values with emphasis on stress concentration patterns; anterior construct stress, measured as peak von Mises stress in the plate-screw construct; posterior cage stress, evaluated separately for ipsilateral and contralateral sides; adjacent segment facet capsule stress at C3/4, C5/6, or C6/7 depending on construct length; and stress distribution patterns in the annulus fibrosus of adjacent discs (C3/4, C5/6, C6/7) with peak stress values recorded.

### 2.6 Data analysis

All simulations were conducted twice to verify the reproducibility. Stress data were obtained from the ANSYS Workbench post-processing tool. The percentage changes relative to the original model were calculated to assess the biomechanical effects of each construct (Hilibrand and Robbins, 2004; Hilibrand et al., 1999). Comparative analyses of ACDF and CCF were conducted at equivalent construct lengths, encompassing one-, two-, and three-level constructs, and also across progressively increasing lengths to discern any length-dependent effects.

### 2.7 Computational resources

The simulations were conducted on a workstation equipped with an Intel Core i7-1470HX processor, a NVIDIA GeForce RTX 4070 Laptop GPU, and a 48 GB RAM memory, along with a 3 TB storage capacity. All seven models under six loading conditions took a total of about 72 h to compute.

## 3 Results

### 3.1 Model validation and mesh convergence

The range of motion (ROM) values for our intact C3–C7 model—4.8° in flexion, 3.9° in extension, 4.2° in lateral bending, and 3.5° in axial rotation—were within one standard deviation of previously reported *in vitro* measurements (Wheeldon et al., 2008; Panjabi et al., 2001), thereby validating the model’s biomechanical accuracy. Specifically, the ROM values in all motion modes differed from the published mean values by less than 12%, falling within the range of inter-specimen variability reported in cadaveric studies. As noted, the von Mises stress variation between consecutive mesh refinements was <5% at 1.5 mm. So, we took that mesh size for all subsequent simulations. Table 2 presents the final counts of elements for each surgical model.

### 3.2 Vertebral body stress distribution chart

**FIGURE 3:**
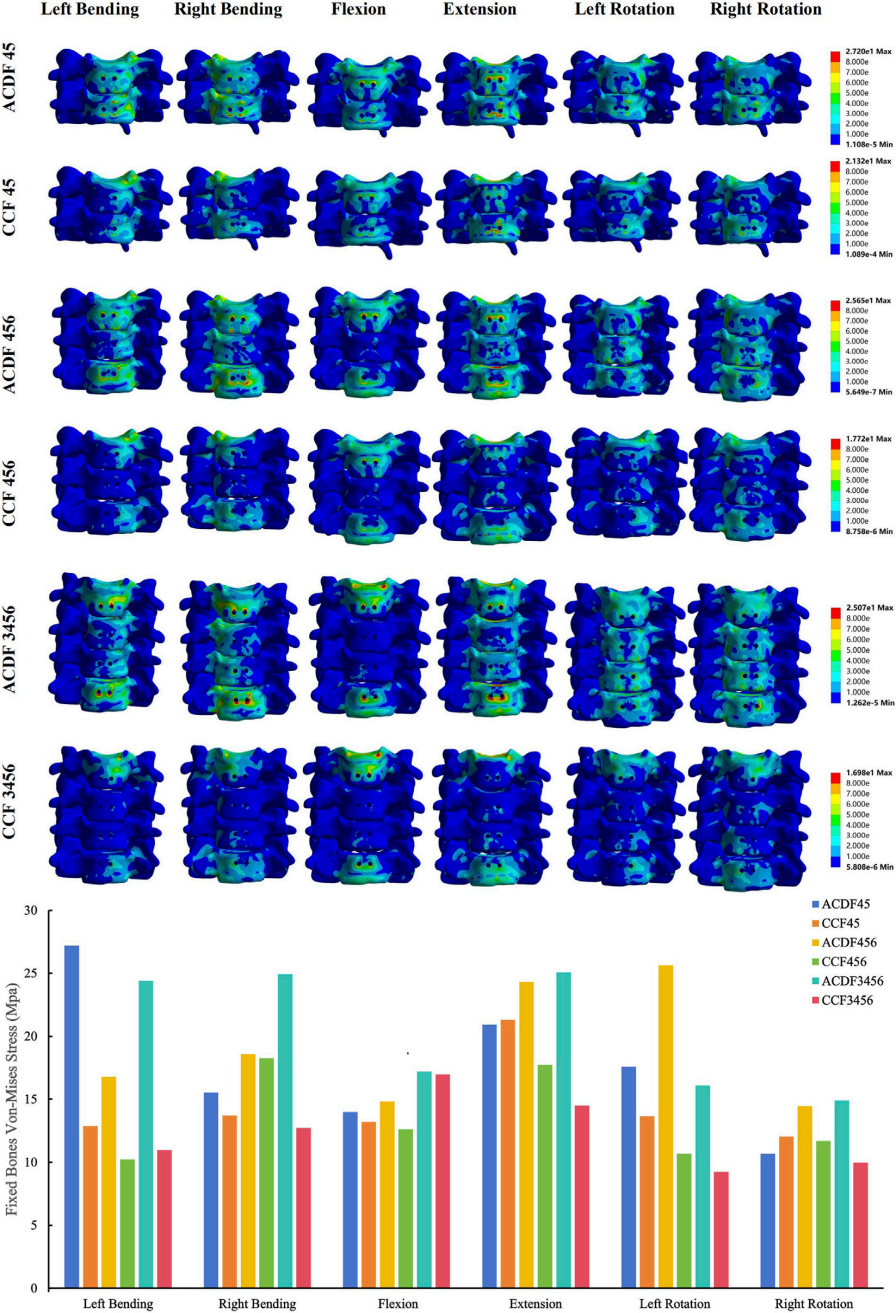
Anterior view of vertebral stress distribution for all models.

**FIGURE 4:**
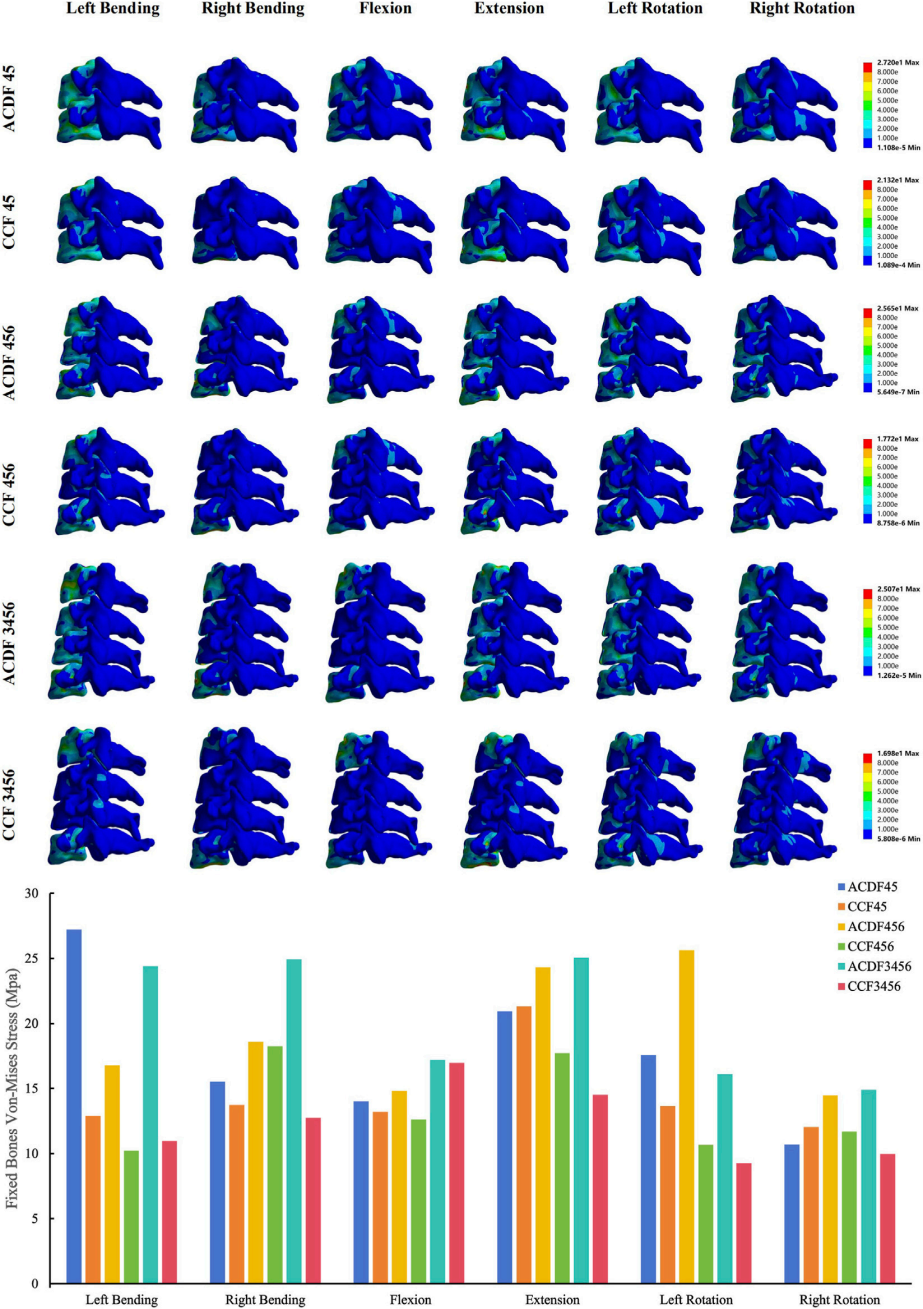
Side view of vertebral stress distribution in fused segments across all models.

In ACDF models, the vertebral body stress decreased as the construct length increased from C4/5 to C4–C6, and then to C3–C6. High stresses were consistently present at the bone–plate–screw interfaces at the cranial and caudal ends of the anterior construct. In all ACDF configurations (Figures 3, 4), the posterior elements of the vertebrae experience minimal stress.

CCF models also demonstrated a length-dependent decrease in vertebral stress, which is distinct from the ACDF model. At the facet–cage interfaces and surrounding posterior cage screw holes, moderate stress was observed. As the construct length increased, the stress-shielding effect of CCF on vertebral bodies became more significant compared to ACDF (Figures 3, 4).

**FIGURE 5:**
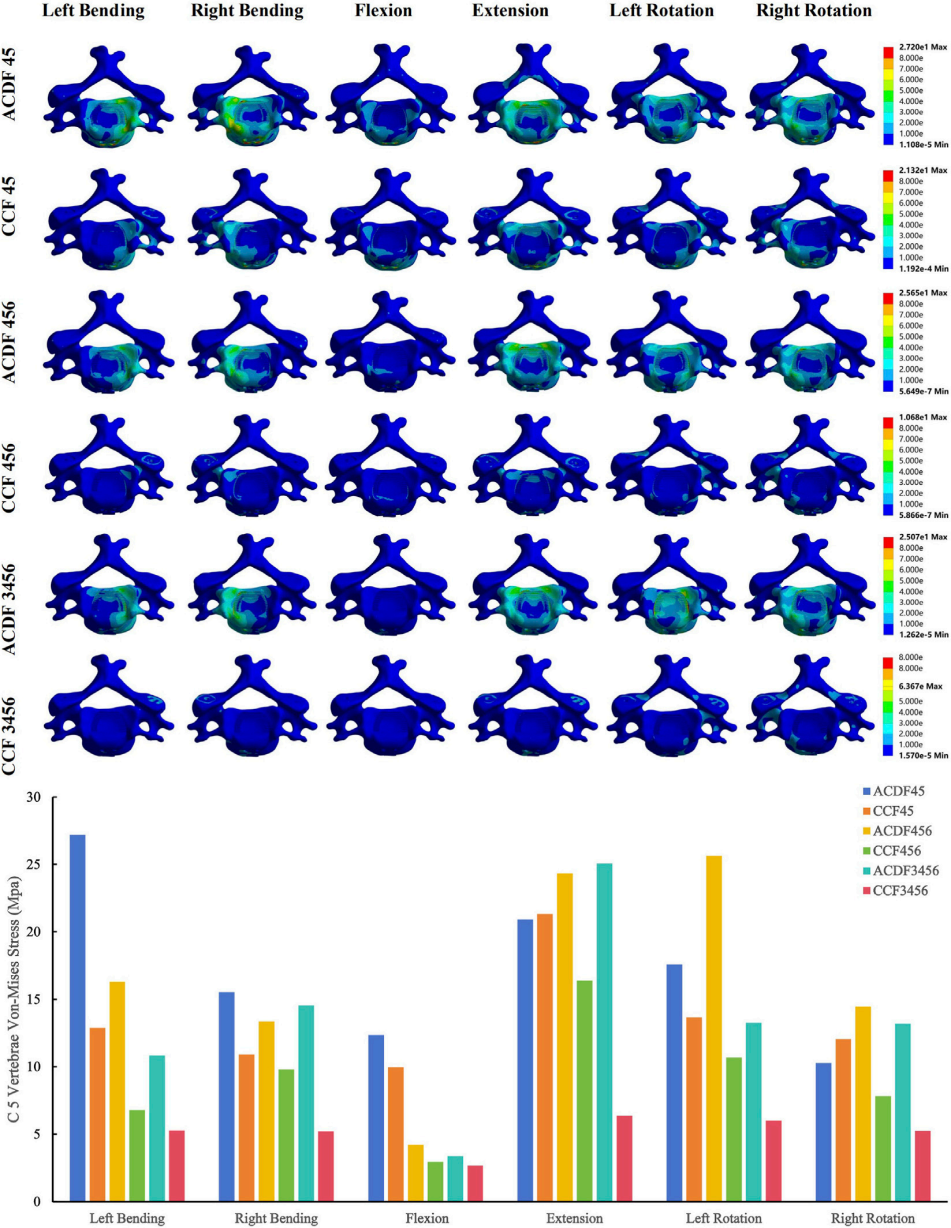
Comparison of C5 vertebral body stress across construct lengths between ACDF and CCF.

Taking C5 vertebral body as a case, CCF models consistently resulted in lower stress compared to ACDF models at the same construct length. The difference was most significant in three-level constructs, where under flexion loading, CCF reduced C5 stress by about 40% compared to ACDF (Figure 5).

## 4 Anterior instrumentation stress

**FIGURE 6:**
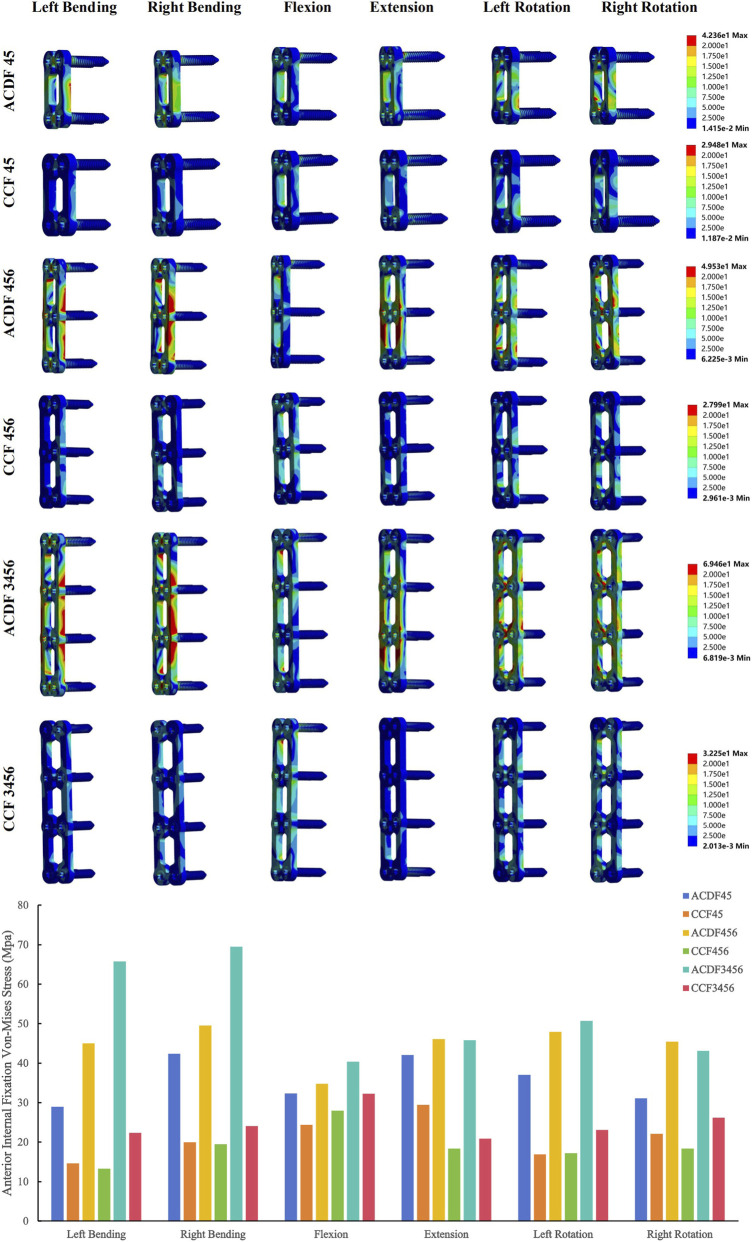
Anterior instrumentation stress comparison across motion modes and construct lengths for ACDF and CCF.

For every motion mode, CCF reduced the anterior instrumentation stress by a significant amount relative to ACDF at the same construct length (Figure 6). The amount of stress reduction increased with the length of the construct: (1) One level: The reduction of all motions by 8%–12% across the level; (2) Two level: 10%–18% reduction; (3) Three level: 15%–25% reduction; (4) The three level constructs experienced a significant drop of 25% during lateral bending.

### 4.1 Posterior instrumentation stress

**FIGURE 7:**
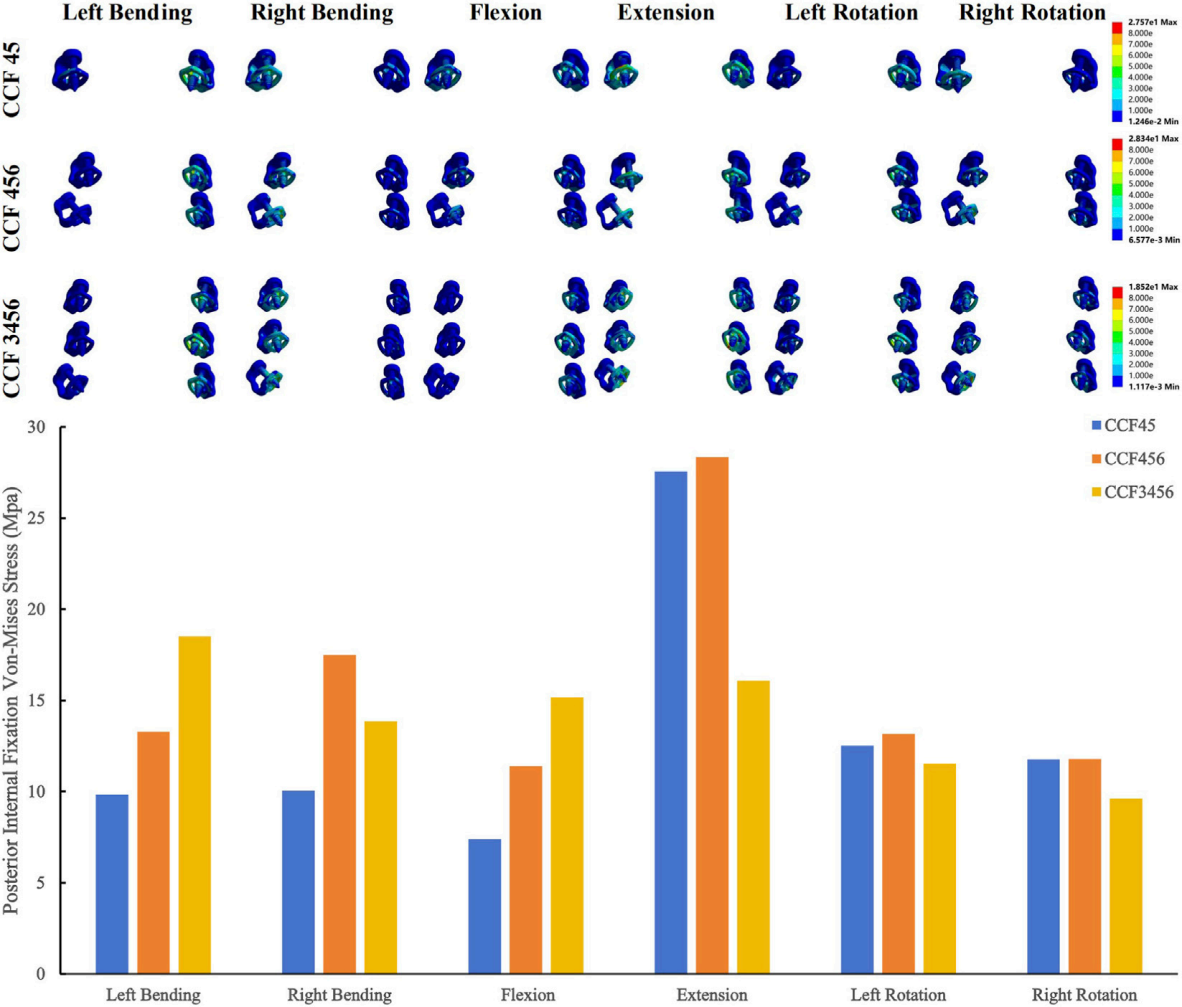
Posterior instrumentation stress in CCF models for motion modes and construct lengths.

Ipsilateral posterior cages have nearly identical stress distributions, so the bilateral implants share load evenly. Among all CCF configurations, three-level CCF had the lowest posterior cage stress (Figure 7). Flexion/extension: Posterior cage stress is significantly lower in flexion compared to extension. During flexion, the cages mainly resisted separation along the screw axis; during extension, they counteract tangential shear forces at the facet joint. Lateral bending: The stress is concentrated on the ipsilateral (bending side) cage, which reflects compressive and shear loading. Axial rotation: Stress was low and unilateral, it resembles lateral bending but has a smaller magnitude.

### 4.2 Adjacent segment facet capsule stress

**FIGURE 8:**
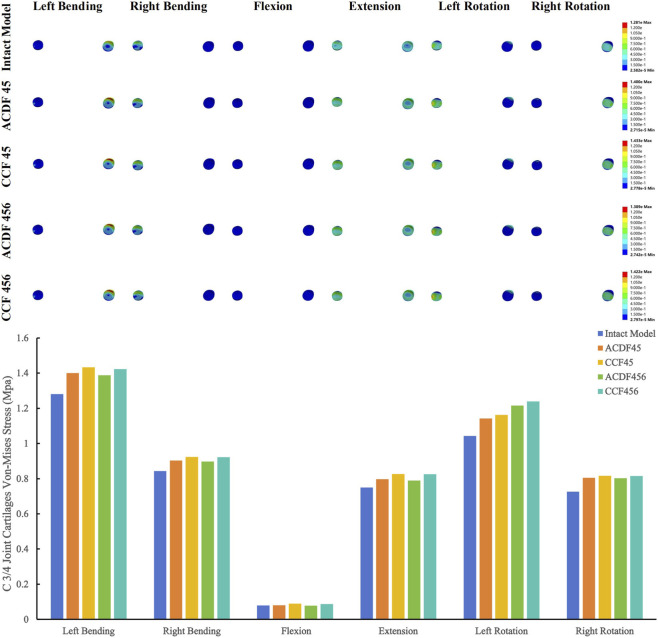
C3/4 facet capsule stress compared to the intact model.

**FIGURE 9:**
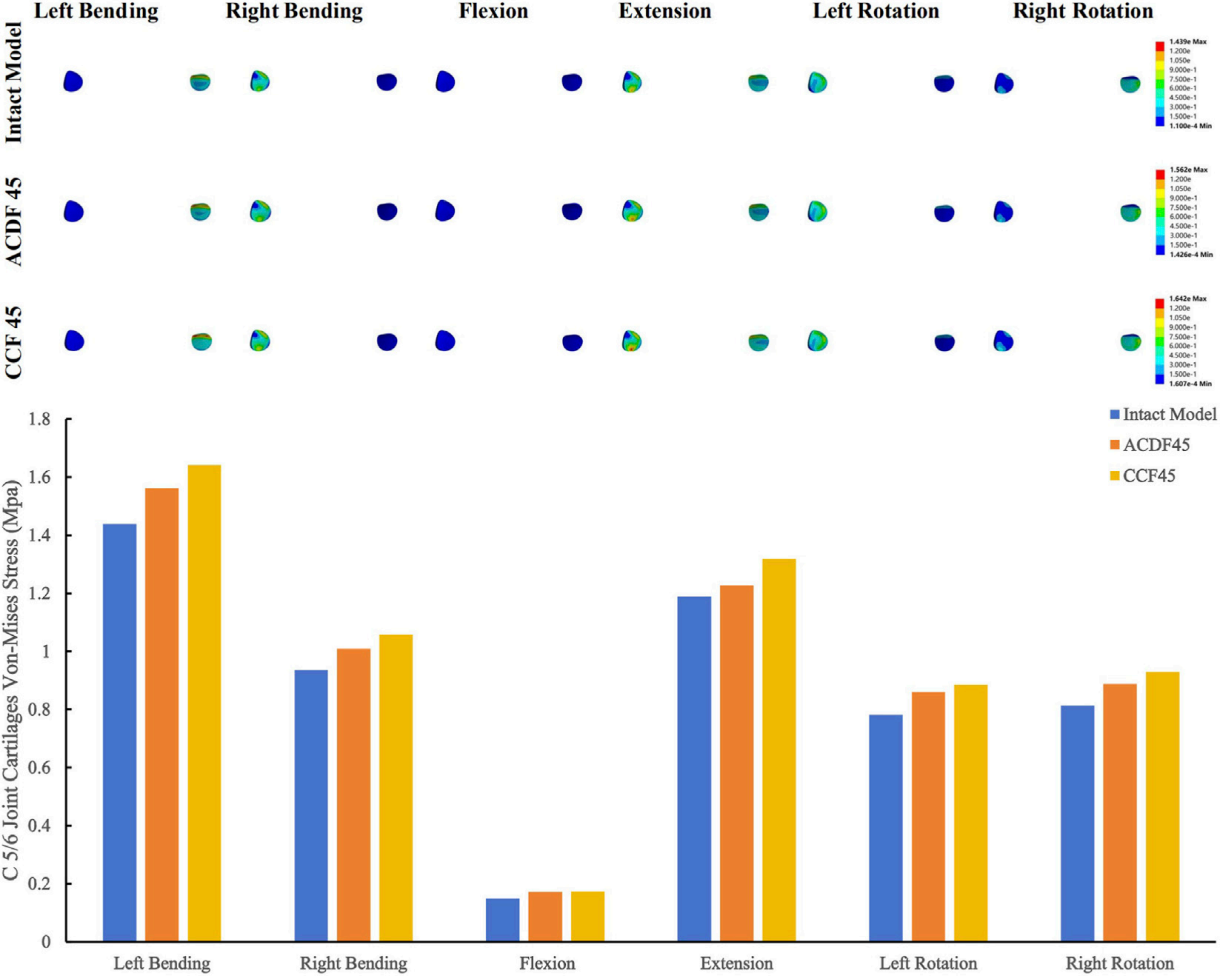
C5/6 facet capsule stress compared to the intact model.

**FIGURE 10:**
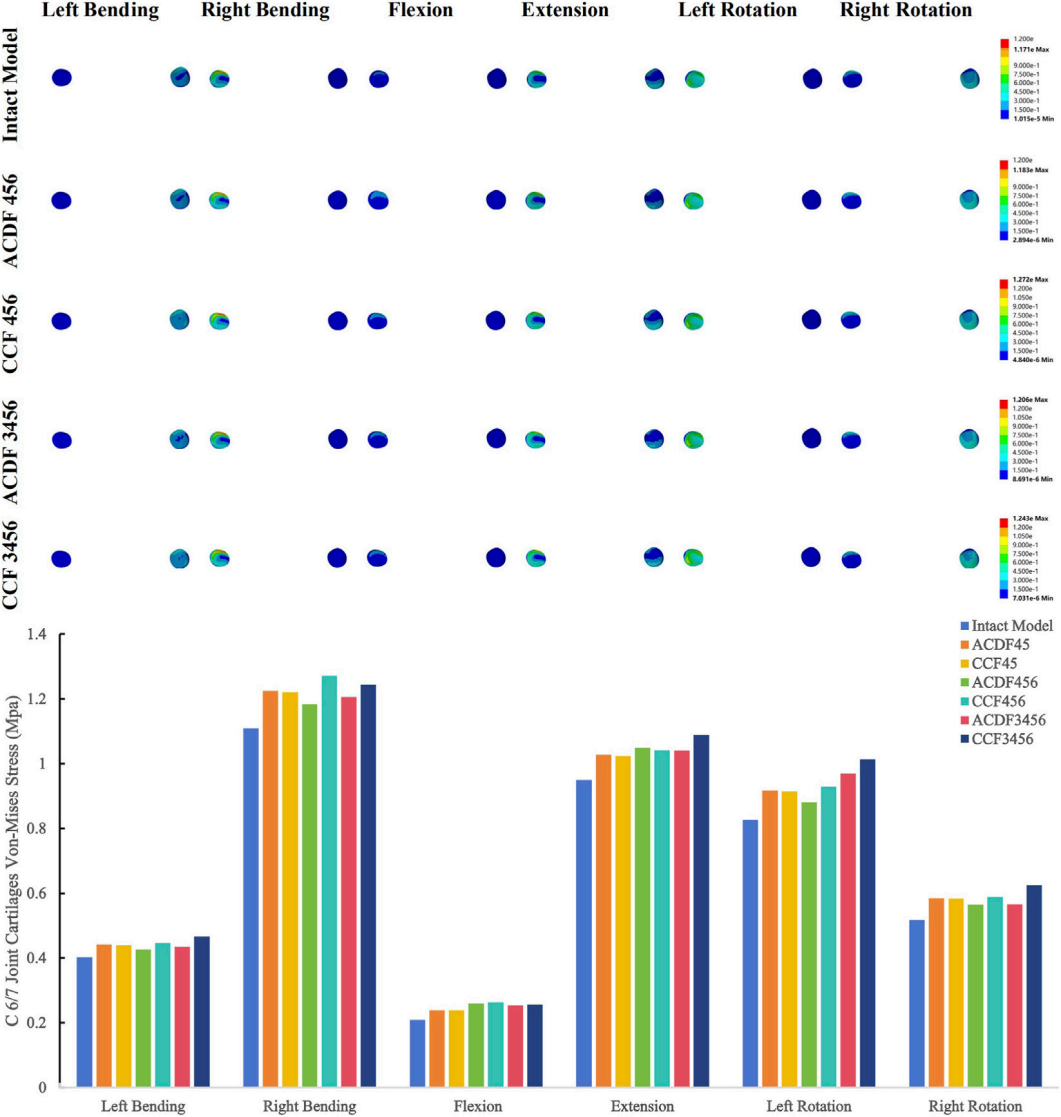
C6/7 facet capsule stress compared to the intact model.

All fusion models have raised the stress at adjacent segments above the intact levels. At equal construct lengths, CCF produced slightly higher facet capsule stress compared to ACDF: (1) C3/4 facet capsule (cranial adjacent segment) (Figure 8): one-level anterior cervical discectomy and fusion (ACDF) increased stress in the C3/4 facet capsule by 9.2% compared to the intact state, while one-level circumferential cervical fusion (CCF) resulted in an 11.9% increase. For two-level constructs, ACDF increased stress by 8.4%, and CCF increased by 1.0%; (2) C5/6 facet capsule (intermediate level) (Figure 9): in one-level constructs (C4/5 fusion), the C5/6 facet capsule served as the caudal adjacent segment. ACDF stress increased by 8.6%, and CCF increased by 14.1%. After two-level fusion, the loss of motion at the treated levels results increased compensatory motion and mechanical stress at the adjacent caudal segment; (3) C6/7 facet capsule (caudal adjacent segment) (Figure 10): for two-level and three-level constructs, the C6/7 facet capsule also experienced stress increase. In the two-level constructs, CCF achieved a 14.6% increase compared to ACDF. In the three-level constructs, CCF achieved a 12.1% increase compared to ACDF; (4) Summary pattern: across all adjacent levels, the stress of facet capsule was consistently higher for CCF compared to ACDF at the same construct length. A significant observation was that facet capsule stress declined with increasing construct length for both methods. Under all loading conditions, the stress distribution patterns within the facet capsule exhibited a general similarity between ACDF and CCF, with the highest stresses consistently recorded during lateral bending.

### 4.3 Adjacent segment intervertebral disc stress

**FIGURE 11:**
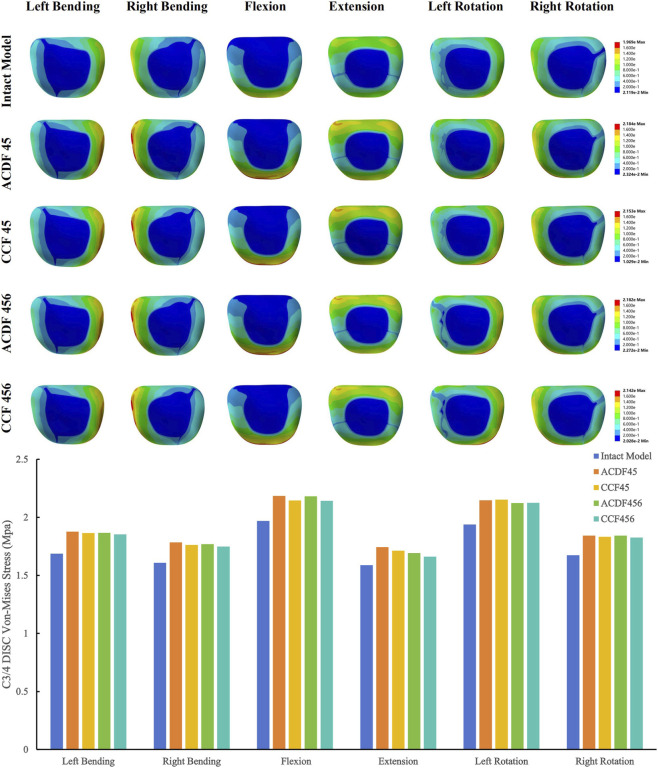
C3/4 disc stress compared to the intact model.

**FIGURE 12:**
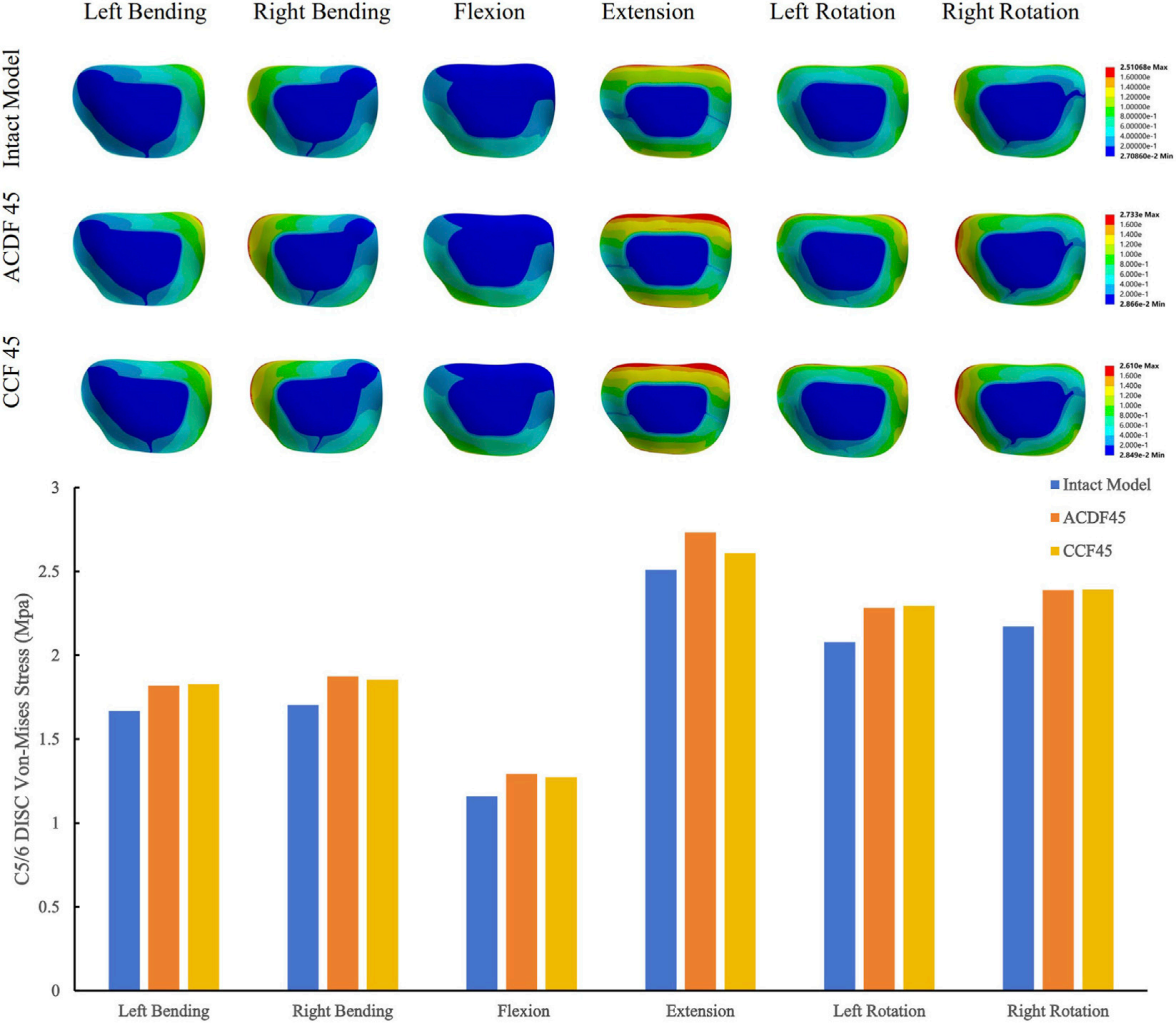
C5/6 disc stress compared to the intact model.

**FIGURE 13:**
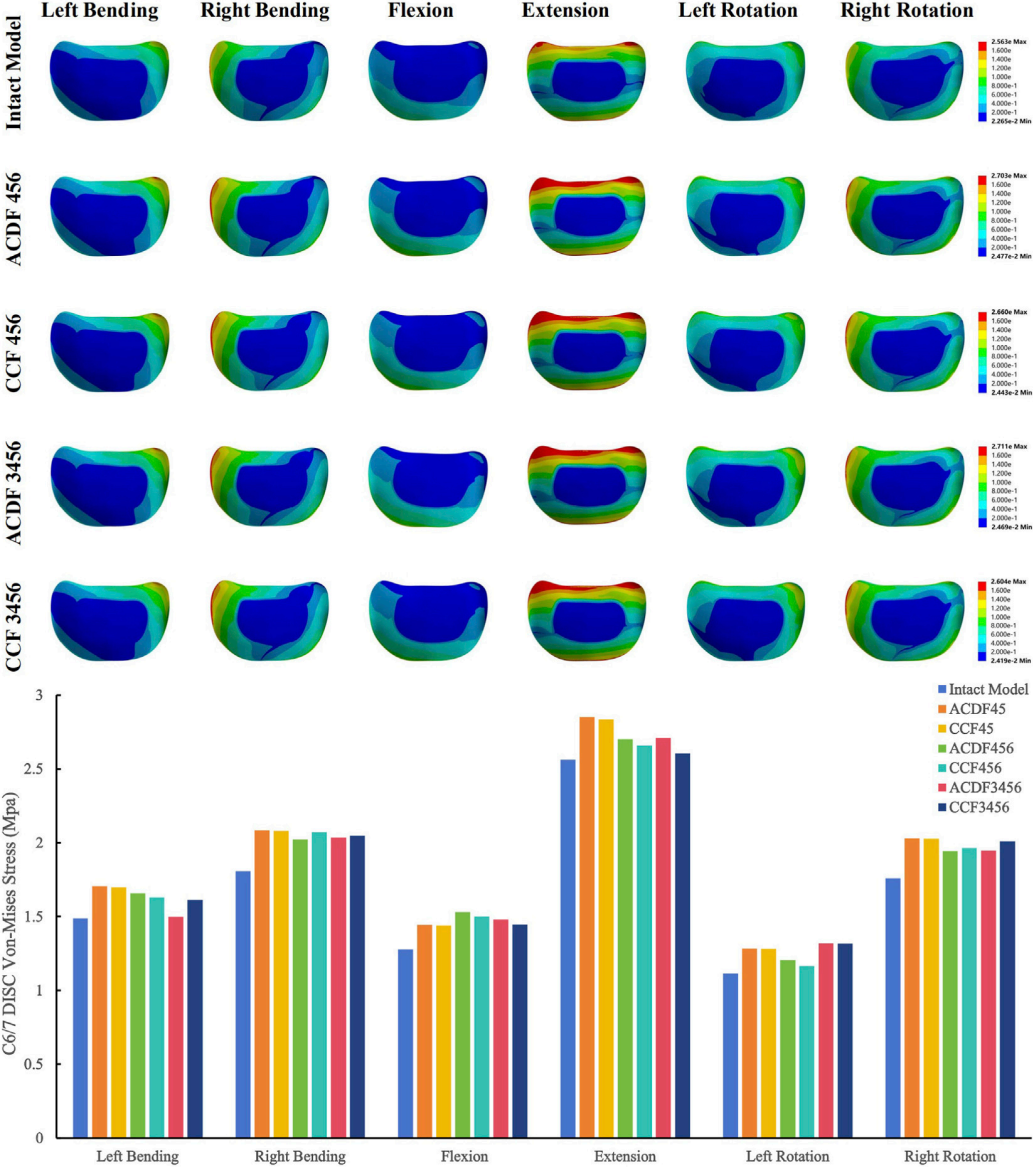
C6/7 disc stress compared to the intact model.

Every fusion model has been raised with disc stress above the intact levels. For every construct length and at each adjacent level analyzed, CCF demonstrated lower stress increases compared to ACDF: (1) C3/4 disc (cranial adjacent segment) (Figure 11): for the C3/4 disc, the ACDF increased stress by 10.9% compared to one-level CCF. In two-level constructs, ACDF increased stress levels by 10.8%, while CCF resulted in an 8.8% rise; (2) C5/6 disc (intermediate level) (Figure 12): in two-level constructs (C4–C6), the C5/6 disc is between the two fused segments, so it was not a true adjacent level. Both ACDF and CCF led to increased stress at the relevant levels, but the increments were less pronounced compared to the cranial adjacent level (C3/4). In three-level constructs (C3–C6), the C5/6 disc was incorporated into the fusion mass and thus excluded from adjacent segment analysis; (3) C6/7 disc (caudal adjacent segment) (Figure 13): for two and three-level constructs, the C6/7 disc exhibited stress elevations. In two-level constructs, ACDF increased its stress by 5.4% and CCF by 3.8%. In three-level constructs, ACDF achieved a 5.8% increase, while CCF only achieved a 1.6% increase, resulting in a 70% reduction in the relative increase compared to ACDF; (4) Stress distribution patterns: in all fusion models, the stress distribution patterns of adjacent discs are characterized by a ring-like distribution, with the maximum stress concentrated at the peripheral annulus on the side aligned with the loading direction. This pattern is consistent in both ACDF and CCF models, and it holds true for all loading conditions.

## 5 Discussion

Our FEA did a systematic comparison of ACDF and CCF for one, two, and three-level cervical constructs. Three main observations were made. First, the biomechanical benefit of adding posterior fixation is determined by the length of the construct, as more segments are fused. Secondly, CCF decreases stress on the anterior spinal construct and redistributes the load to the posterior elements, thus reducing the likelihood of anterior hardware failure and pseudarthrosis. Third, the effects on adjacent segments are mixed: CCF raises facet capsule stress relative to ACDF, but reduces adjacent disc stress, especially in longer constructs. The findings provide a mechanistic foundation for the clinical outcomes reported by Strenge et al. (2025) and serve as a valuable reference for selecting surgical approaches based on varying construct lengths.

### 5.1 Biomechanical effects influenced by length

A distinct correlation between construct length and biomechanical performance was identified. For one-level constructs, ACDF alone provided a stable and favorable anterior stress distribution (Hua et al., 2020). The addition of posterior fixation offers minimal additional biomechanical advantage, aligning with the established clinical success of single-level anterior cervical discectomy and fusion (ACDF). This finding corroborates the prevailing practice of seldom utilizing supplementary posterior stabilization for single-level spinal conditions (Halder et al., 2026).

For two-level constructs, ACDF models started to exhibit biomechanical weaknesses (Chien et al., 2016). Anterior cage stress was higher than one-level constructs, especially at the caudal level. CCF began to provide protection, which reduces the anterior cage stress by 10%–18% across all motions. This suggests that although two-level anterior cervical discectomy and fusion (ACDF) is biomechanically suitable for many patients, incorporating posterior fixation may benefit those with risk factors for pseudarthrosis, including smoking, osteoporosis, or a high body mass index.

For three-level constructs, ACDF only showed a significant biomechanical compromise. Anterior cage stress was significantly increased, with a strong emphasis at the caudal screw–bone interface. These findings align with clinical reports indicating that three-level anterior cervical discectomy and fusion (ACDF) exhibits high nonunion rates, especially at the caudal level (Jang et al., 2022; Lee et al., 2018). The use of circumferential cervical fusion (CCF) significantly enhanced the biomechanical environment by reducing anterior cage stress by 15%–25% and redistributing loads through the posterior facet cages. The most significant protective effect occurred during lateral bending, a movement recognized for generating substantial shear forces on the anterior spinal construct (Voronov et al., 2016; Jin et al., 2021). These findings provide a mechanistic explanation for the improved fusion and lower revision rates observed in the CCF group within the randomized trial (Strenge et al., 2025).

### 5.2 Mechanisms of stress shielding in posterior fixation

The stress patterns indicate how CCF safeguards the construct. In ACDF models, stress is concentrated mainly in the anterior column, and there is very little load passing through the posterior elements. This anterior-dominant pathway causes plate-screw construct and interbody cage to be subjected to high cyclic stresses, increasing the risk of hardware failure and pseudarthrosis (Peterson et al., 2018).

And CCF models got a more balanced load distribution. The posterior facet cages bore a significant portion of the applied load, particularly during extension and lateral bending movements. Nearly the same stress on ipsilateral cages suggests that the load sharing is effective, and the posterior construct works an integrated unit. Posterior cage stress was notably lowest in three-level constructs, suggesting that longer constructs may facilitate a more advantageous load-sharing distribution, likely due to the dispersion of loads across a broader area by multiple fixation points.

These findings align with earlier biomechanical studies indicating that circumferential fusion reduces strain on the anterior construct, which is less than ACDF alone (Pereira et al., 2021; Li et al., 2021). However, our work further demonstrates that this protective effect is not just “present or absent” but depends on the length of the construct. The anterior stress decreases as more posterior fixation points are added, which supports the idea that supplemental posterior stabilization should be seen as a length-dependent intervention.

### 5.3 Adjacent segment effects: a detailed analysis

The risk of adjacent segment degeneration (ASD) after cervical fusion is well know, so the effects on adjacent segments are clinically important (Carrier et al., 2013; Chung et al., 2016). Our analysis revealed that ACDF and CCF exert varying influences on adjacent structures.

For facet capsules, CCF consistently produced larger stress increases compared to ACDF at the same construct length. This implies that posterior fixation causes additional load to be transmitted to the adjacent facet joints, potentially increasing the likelihood of symptomatic ASD. Meanwhile, we also found out that the stress from facet capsule decreased as the construct length increased, suggesting that longer fusions might actually protect the immediate adjacent facet capsules by distributing motion demands over a longer spinal segment. This length-dependent effect requires further investigation in long-term clinical trials.

The pattern was reversed for intervertebral discs. CCF consistently provides lower adjacent disc stress compared to ACDF at the same construct length. The most significant difference was in the caudal adjacent disc, where CCF had a 1.6% increase compared to ACDF. So, posterior fixation might reduce the kinematic load on adjacent discs, which could lower the risk of disc-related ASD. The differences in the effects on facet capsules and discs show the complexity of adjacent segment biomechanics, and we need a comprehensive assessment when comparing fusion techniques.

The observed ring-like stress pattern in adjacent discs, characterized by peak stress at the peripheral annulus on the loaded side, aligns with findings from previous finite element analysis (FEA) studies and demonstrates the intervertebral disc’s response to altered load transmission following fusion (Ardatov et al., 2026; Umale and Yoganandan, 2018).

The observed trade-off—higher facet capsule stress but lower disc stress with CCF compared to ACDF—carries potential long-term clinical implications. Elevated facet capsule stress may predispose patients to symptomatic facet arthrosis, potentially manifesting as axial neck pain or limited range of motion over time (Li et al., 2024). Conversely, the reduced stress on adjacent discs suggests a possible protective effect against disc-related adjacent segment degeneration, such as herniation or progressive disc collapse. In clinical practice, this biomechanical profile suggests that patient-specific factors should guide surgical selection: for patients with pre-existing facet degeneration or predominant axial symptoms, the additional facet loading from CCF may be less desirable; for those with patent facet joints but high-risk disc pathology, CCF’s protective effect on adjacent discs may be advantageous. However, these biomechanical trends require validation through long-term clinical studies correlating these stress parameters with symptomatic ASD rates.

### 5.4 Comparison with clinical studies

Our biomechanical results match the clinical trial results of Strenge et al. (Strenge et al., 2025) In that RCT, three-level ACDF alone resulted in a 12-month fusion rate of 17% and a 24-month revision rate of 23%, while CCF achieved 61% fusion and 2% revision. The observation that three-level anterior cervical discectomy and fusion (ACDF) exhibits significant anterior construct stress concentration, particularly in the caudal region, offers a mechanistic insight into the clinically observed high rates of pseudarthrosis. Our observation indicates that CCF effectively redistributes load and reduces anterior stress, which corroborates the clinical finding that incorporating posterior fixation enhances fusion results.

Our study provides a mechanistic explanation for the trial result, indicating that 1 out of 13 revisions in the ACDF arm were for symptomatic nonunion. The stress concentration at the caudal level that we identified aligns with clinical observations indicating that this level is most susceptible to failure in multilevel Anterior Cervical Discectomy and Fusion (ACDF) procedures. Demonstrating that circumferential cervical fusion (CCF) effectively reduces stress at the caudal level supports this clinical pattern.

The absence of increased adverse events in the CCF arm is supported by our finding that CCF does not generate excessive stress concentrations that would cause hardware failure. The ipsilateral posterior cages have a robust design for even load sharing, which reduces implant fatigue or failure risk.

### 5.5 Limitations

Several limitations are necessary to be acknowledged. First, FEA is a computational model that is idealized, and it can’t fully capture the biological complexity of bone healing, patient-to-patient anatomical differences, and the dynamic effects of muscle forces and postoperative loading (Varga et al., 2020). We validated our model against published data, but the results should be viewed as biomechanical trends, not absolute clinical predictions.

Second, our model assumed that postoperative conditions are immediate and there is no bone healing. As fusion continues to develop and bone bridges form across the treated areas, the biomechanical environment changes a lot (Hu et al., 2019). Future studies with time-dependent bone remodeling and fusion progression will provide insights.

Third, we employed linear elastic and isotropic material properties, which simplify the anisotropic and viscoelastic behaviors of living tissues. But this approach is standard in comparative FEA, and it enables consistent comparisons across different surgical conditions.

Fourth, the analysis employed a geometry derived from a single healthy adult male. This approach eliminates inter-subject anatomical variability, enabling a controlled parametric comparison of surgical constructs. However, it does not capture the range of patient anatomies, bone mineral densities, or degenerative changes encountered in clinical practice. Cervical spine morphology varies significantly with age, sex, and pathology; thus, the absolute stress values reported here may not directly apply to patients with osteoporosis, severe osteophyte formation, or altered cervical alignment. Nevertheless, the relative trends between ACDF and CCF across construct lengths—specifically the length-dependent stress shielding and load redistribution—are likely to be qualitatively generalizable, as they are governed by fundamental biomechanical principles of load path and moment arm. Future studies employing multiple anatomical models or parametric variations in bone quality and sagittal alignment would enhance the generalizability of these findings.

Fifthly, the posterior fixation system modeled in this study is a specific investigational device known as PCSS. The results validate its biomechanical efficacy; however, these findings may not extend to other posterior fixation techniques, such as conventional lateral mass screw-rod systems.

Finally, although we found differential effects on facet capsule and disc stress, the clinical implications of these findings are still unknown. Long-term clinical studies are need to link these biomechanical parameters to symptomatic ASD rates.

### 5.6 Clinical implications

Our findings have several practical applications. First, single-level ACDF alone offers adequate biomechanical stability, therefore, routine posterior augmentation is not necessary. Second, for two-level constructs, surgeons should consider patient-specific risk factors when deciding whether to add posterior fixation. Thirdly, when dealing with three-level constructs, our biomechanical data provide robust evidence for the use of supplemental posterior fixation. This approach effectively reduces stress on the anterior construct and enhances the probability of achieving successful fusion. Fourth, the trade-off—higher facet capsule stress but lower disc stress with CCF—should be considered from the perspective of each patient’s anatomy and pathology.

### 5.7 Future directions

A lot of further research avenues are emerging. Parametric studies on bone quality, implant material, and screw configuration might improve the selection of patients for CCF. Dynamic FEA with muscle forces and physiological loading patterns would give more understanding of the time-dependent biomechanical environment (Hume et al., 2020). Integrating computational and clinical research to link biomechanical parameters with fusion success rates and adjacent segment degeneration (ASD) rates enhances the practical applicability of the findings. Finally, cost-effectiveness analyses that account for the reduced revision rates with CCF could guide healthcare policy and resource allocation.

## 6 Conclusion

This finite element analysis indicates that the biomechanical benefit of adding posterior fixation in cervical fusion relies on the construct length. For one-level constructs, ACDF alone is sufficient for stability. For two-level constructs, the biomechanical weakness supports the use of posterior fixation in high-risk patients. For three-level constructs, CCF provides a lot of biomechanical advantages by reducing anterior construct stress, redistributing loads, and enhancing stability. Though CCF causes the adjacent facet capsule stress to be relative to ACDF, it also reduces the adjacent disc stress, resulting in a more complex biomechanical profile. The results offer mechanistic backing for the outcomes of recent randomized controlled trials and serve as a valuable reference for surgical decision-making in patients suffering from multilevel cervical degenerative disease.

## Data Availability

The original contributions presented in the study are included in the article/supplementary material, further inquiries can be directed to the corresponding author.
